# A first look at the ability to use genomic prediction for improving the ratooning ability of sugarcane

**DOI:** 10.3389/fpls.2023.1205999

**Published:** 2023-08-02

**Authors:** Md. Sariful Islam, Keo Corak, Per McCord, Amanda M. Hulse-Kemp, Alexander E. Lipka

**Affiliations:** ^1^ Sugarcane Field Station, USDA-ARS, Canal Point, FL, United States; ^2^ Genomics and Bioinformatics Research Unit, USDA-ARS, Raleigh, NC, United States; ^3^ Irrigated Agriculture Research and Extension Center, Washington State University, Prosser, WA, United States; ^4^ Department of Crop and Soil Sciences, North Carolina State University, Raleigh, NC, United States; ^5^ Department of Crop Sciences, University of Illinois, Urbana-Champaign, IL, United States

**Keywords:** candidate gene, GWAS, genomic selection, marker trait association, sugarcane production

## Abstract

The sugarcane ratooning ability (RA) is the most important target trait for breeders seeking to enhance the profitability of sugarcane production by reducing the planting cost. Understanding the genetics governing the RA could help breeders by identifying molecular markers that could be used for genomics-assisted breeding (GAB). A replicated field trial was conducted for three crop cycles (plant cane, first ratoon, and second ratoon) using 432 sugarcane clones and used for conducting genome-wide association and genomic prediction of five sugar and yield component traits of the RA. The RA traits for economic index (EI), stalk population (SP), stalk weight (SW), tonns of cane per hectare (TCH), and tonns of sucrose per hectare (TSH) were estimated from the yield and sugar data. A total of six putative quantitative trait loci and eight nonredundant single-nucleotide polymorphism (SNP) markers were associated with all five tested RA traits and appear to be unique. Seven putative candidate genes were colocated with significant SNPs associated with the five RA traits. The genomic prediction accuracies for those tested traits were moderate and ranged from 0.21 to 0.36. However, the models fitting fixed effects for the most significant associated markers for each respective trait did not give any advantages over the standard models without fixed effects. As a result of this study, more robust markers could be used in the future for clone selection in sugarcane, potentially helping resolve the genetic control of the RA in sugarcane.

## 1 Introduction

Sugarcane (*Saccharum* sp. hybrid) production is vital to the agricultural economy of tropical and subtropical regions, where 86% of the world’s table sugar is produced (FAO, 2020). Sugarcane is also used in the production of bioethanol, energy from the bagasse (sugar production by-product), and paper products. Sugarcane is commonly managed through an agronomic practice known as ratooning in which growers plant the sugarcane once and then harvest the aboveground biomass several times, allowing the plant to recover between harvests. The first-year crop grown from the seed cane after planting is called the plant cane (PC) crop. The subsequent regrowth from the stubble after harvesting is called the ratoon (RT) crop. RT crops can be collected multiple times. In Florida, during the 2020 growing season, 69.8% of the sugarcane cultivation area was under RT crops composed of the first ratoon (FR; 30.5%), second ratoon (SR; 29.5%), third ratoon (7.6%), and fourth ratoon (2.3%) (VanWeelden et al., 2020). Depending on the growing region, the proportion of RT crops ranges from 50% to 75% of the total sugarcane cultivated area in other countries (Xu et al., 2021). Like in every other sector, agricultural producers are struggling with the high cost of labor and associated labor shortages all over the world. This problem is especially acute in western countries like the United States. Ratooning saves growers’ money on cultivation costs, and it boosts industry profits because RT crops mature earlier, produce better juice quality, and have better sugar recovery (Xu et al., 2021). Thus, by increasing the proportion of acreage in ratoon crops, sugarcane industries can save on labor costs, increase sugar quality, and decrease soil disturbance by skipping planting operations every year.

Unfortunately, sugarcane yield tends to decline after the first PC crop (Xu et al., 2021). Therefore, sugarcane breeders are currently selecting for a trait they have described as the ratooning ability (RA) in addition to other desired traits in their breeding objectives to make the sugarcane industry more profitable. The RA has various definitions; the most common definition expresses the performance of RT crops (the first ratoon or the average of all ratoons) as a percentage relative to the PC crop as described (Equation 3) below (Milligan et al., 1996; Coto Arbelo et al., 2021). Industry stakeholders desire varietals that can be grown over an increased number of ratoon crops while maintaining their yield. Performance is determined by several morphological and yield traits including root growth of stubble, germination rate after harvesting, tillering rate, stalk number in previous crop, and cane yield, which is a product of stalk population (SP) and stalk weight (SW) (Milligan et al., 1990). Thus, selecting on those traits will generally improve the RA. However, the RA of sugarcane is not determined just by genetics but by a combination of factors including environment (soil, temperature, humidity, and water supply), cultivation technology (Xu et al., 2021), and the interactions between them. Several studies have been reported related to the RA in context of the environment, biotic and abiotic stress, and management practices (Singh et al., 2006; Shukla et al., 2008; Gomathi et al., 2013; Ramburan et al., 2013; Bagyalakshmi et al., 2019; Gao et al., 2019).

Very little is known about the genetics and genetic architecture of RA in sugarcane (Milligan et al., 1996; Zhou and Shoko, 2012; Coto Arbelo et al., 2021). The most cited report on the RA studied the heritability, correlation between PC and RT crop, and genetic gain in RT crop of six sugar- and yield-related traits using 37 genotypes (Milligan et al., 1996). Another study suggested that the high-yielding genotypes with slightly lower RAs might be viable for commercial production when planted in short ratooning cycles, since higher SP seems to diminish the yield loss in ratoon crop (Zhou and Shoko, 2012). Very recently, although a narrow RA diversity was detected among the 39 tested clones, three checks using the same strategy recommended two clones for commercial release having high yield and good RA potential in a multilocation trial (Coto Arbelo et al., 2021). To the best of our knowledge, no report has been published related to the identification of quantitative trait loci (QTLs) or marker–trait associations for the RA in sugarcane. Investigating the genetic and molecular basis of the RA using a genome-wide association study (GWAS) in sugarcane will assist breeders in better understanding the genetic basis for potentially improving the RA of sugarcane cultivars.

GWASs are widely used to identify genes, mutations, and putative functional markers that are responsible for complex quantitative traits through forward genetics analysis (Vuong et al., 2015; Islam et al., 2016). In comparison with traditional QTL analyses, GWAS has several advantages, including using materials with increased diversity and better downstream utility across diverse germplasms with a shorter development time for new populations (Abdurakhmonov and Abdukarimov, 2008). Thus, GWAS has been successfully utilized in many plants for identifying significant marker–trait associations and putative candidate genes, such as cotton (Islam et al., 2016; Thyssen et al., 2018; Wubben et al., 2019), maize (Pace et al., 2015), barley (Matthies et al., 2014), wheat (Tadesse et al., 2015), sorghum (Morris et al., 2013), and soybean (Vuong et al., 2015). However, many genetic studies using GWAS have been reported in sugarcane (Debibakas et al., 2014; Racedo et al., 2016; Singh et al., 2016; Barreto et al., 2019; Yang et al., 2019a; Yang et al., 2019b; Yang et al., 2020; Pimenta et al., 2021; Chen et al., 2022). To the best of our knowledge, there have been no QTL and/or GWASs related to the RA trait in sugarcane reported so far. Hence, conducting a GWAS to detect markers associated with the RA in sugarcane would be an important contribution to the sugarcane-breeding community. Discovered significant genetic loci could be incorporated into a breeding pipeline, but marker-assisted selection (MAS) approaches have several limitations, for example, many traits are polygenic in nature with small QTL effects and large genotype-by-environment interactions (Xu and Crouch, 2008).

In addition to the insights gained from GWAS approaches, breeders are interested in using genome-wide genotypic data to estimate the genomic estimated breeding value (GEBV) of testing individuals (Meuwissen, 2009) and to use these GEBVs for selection. Numerous animal- and plant-breeding programs have already used genomic selection (GS) successfully since its introduction. Unlike MAS, which typically only considers major effect loci, the use of genome-wide markers allows GS to account for both major and minor effect loci (Gouy et al., 2013). A breeding cycle could be shortened if individuals were selected based on their GEBV in the early phases of the breeding cycle (Jannink et al., 2010). In sugarcane specifically, predicting the RA could allow breeders to potentially shorten the breeding cycle by reducing ratoon crop cycles during selection.

The utility of GS has increased as new bioinformatics tools and next-generation sequencing have been developed. Thus, GS has been evaluated in many crops (Arruda et al., 2015; Bernal-Vasquez et al., 2017; Gezan et al., 2017; Islam et al., 2020) including 11 studies in sugarcane (Gouy et al., 2013; Olatoye et al., 2019; Deomano et al., 2020; Hayes et al., 2021; Islam et al., 2021; Voss-Fels et al., 2021; Yadav et al., 2021; Aono et al., 2022; Batista et al., 2022; Islam et al., 2022a; O’Connell et al., 2022). However, none of the studies attempted to predict the RA in sugarcane. Hence, it is worthwhile to conduct research on evaluating the genomic prediction accuracy of the RA in sugarcane. It is reported that incorporating major gene or QTL in the GS models as fixed covariates could increase the prediction accuracy most of the time (Bernardo, 2014; Islam et al., 2022a). However, it is not true all the time: it has also been reported that prediction accuracy was decreased and model bias was increased by incorporating the GWAS peak marker as a fixed effect in the GS models depending on the genetic architecture of the trait (Rice and Lipka, 2019; Billings et al., 2022).

We have previously reported two GS studies related to disease resistance, sugar, and yield-related traits using the same population and the same sets of markers as in the current study (Islam et al., 2021; Islam et al., 2022a). The objective of this study is to uncover the genetic basis of the RA and detect the associated markers with five RA traits in sugarcane. We also evaluated four GS models for checking the feasibility of GS in the RA trait in sugarcane using the peak GWAS marker as a fixed covariate in the tested models. This research contributes to understanding the genetic basis of the RA traits, developing more robust trait-associated markers, along with providing information about using the GS approach for sugarcane clone selection and downstream cultivar release to growers.

## 2 Materials and methods

### 2.1 Plant material and field trial

Four hundred fourteen sugarcane clones from the second clonal stage of the USDA-ARS Sugarcane Breeding Program at Canal Point, Florida, program and 18 commercial cultivars from the USDA-ARS Sugarcane Breeding Program in Houma, Louisiana, were grown in a replicated field trial (**Supplementary Table S1**) along with two checks, “CP 00-1101” (Gilbert et al., 2008) and “CP 96-1252” (Edmé et al., 2005). Each tested clone was replicated twice in an augmented row–column experimental design, and each check was repeated 17 times. The details and layout of the field plots are described in our previous studies (Islam et al., 2021; Islam et al., 2022a). In brief, the field trial was established at the USDA-ARS Sugarcane Field Station, Canal Point, Florida, in November 2016. Plots consist of one row (4.6 m in length) with 1.5-m spacing between plots. Each row had 25 plots with an alley between two rows of 6 m between each row. The field was divided into 36 rows. Three crop cycles were evaluated: PC, FR, and SR. The standard protocol was followed for all necessary management practices throughout the trial.

### 2.2 Data collection and trait measurement

The details of data collection of yield and sugar traits were described in our previous reports (Islam et al., 2022a). SP, SW, total dissolved solids (Brix), juice polarization (Pol), and fiber content data were collected from the field plot directly for PC, FR, and SR crop cycles. In September 2017, 2018, and 2019, the millable stalks per hectare (SP) for each plot were estimated from the number of stalks per plot counted manually for PC, FR, and SR, respectively. We harvested 10 stalks from each plot randomly, removed the top just below the apical meristem, and bundled each plot in February 2018, 2019, and 2020 for PC, FR, and SR, respectively. Those harvested bundles were utilized to collect SW (kg stalk^-1^), total fiber content (%), Pol (%), Brix (%), and moisture content (%). Sugar content (SC) was then calculated from the corrected Brix and Pol (Legendre, 1992) using the following formula (Islam et al., 2022b):


(1) $$SC\;(\%)=\frac{Pol\times 26}{\left[105.811+(Brix-15)\times 0.444\right]}$$

Cane yield (Mg ha^-1^) in the form of tonns of cane per hectare (TCH) was calculated as the product of SW (kg stalk^-1^) and SP (stalks ha^-1^) and divided by 1,000. Theoretical recoverable sucrose (TRS) was calculated from the juice data and fiber concentration to estimate the sugar yield (Legendre, 1992). All values of TRS were multiplied by a correction factor of 0.86 to approximate the commercial recoverable sugar (CRS; kg ha^-1^) as suggested by Islam et al. (2022b). Sucrose yield (Mg ha^-1^) as tonns of sucrose per hectare (TSH) was estimated as:


(2) Sucrose yield = (TCH×CRS)÷1000

Following Deren et al. (1995), the economic index (EI) was calculated from the cane yield, sucrose yield, and costs of harvesting, hauling, and milling the cane in Florida. The RA (%) for EI, TCH, TSH, SP, and SW was then estimated as suggested by Coto Arbelo et al. (2021) and Milligan et al. (1996) using the following formula:


(3) $$A=\frac{\sum_{n=1}^{N}R_{n}}{P*N}*100$$

where A is the RA for a given trait expressed as a percent, *R_n_* is the phenotypic value of the trait in RT crop *n*, P is the value of the trait in the PC crop, and N is the number of ratoon crops.

### 2.3 Genotyping and single-nucleotide polymorphism markers

DNA extraction, library preparation, sequencing, single-nucleotide polymorphism (SNP) calling, and filtering protocols were described in detail in our previous reports (Islam et al., 2021; Islam et al., 2022a). After extracting, DNA from young leaves was submitted to RAPiD Genomics LLC (Gainesville, FL, USA) for library preparation, sequencing, and initial bioinformatic analysis. Sequencing was done on an Illumina HiSeq 2 X 100 sequencer with the qualified processed samples combined in equal amounts. The raw sequence data were demultiplexed using Illumina’s bcl2fastq, and then reads were cleaned and trimmed using the FASTX-Toolkit (http://hannonlab.cshl.edu/fastx_toolkit/index.html). Mosaik (Lee et al., 2014) was used to align clean reads against the *Sorghum bicolor* V3.1 reference genome (Paterson et al., 2009). Using FreeBayes (Garrison and Marth, 2012), the SNPs were called and then filtered based on read depth for each SNP ≥35 and minor allele frequency ≥2%. Because of the conversion, marker data were transformed into numerical format, where 0 was assigned to the reference allele at each locus, 2 to the alternate allele, and 1 to the heterozygote. After discarding SNPs with more than two alleles, 10,435 SNPs were remaining for further analysis. The SNP nomenclature was given starting with sorghum reference genome chromosome number followed by the location in the chromosome.

### 2.4 Variance component, heritability, and correlation

The best linear unbiased predictors (BLUPs) of the RA of the five tested traits for the sugarcane clones were analyzed using a single mixed linear model as described below:


(4) $$y_{aikhj}=\mu +p_{j}+g_{a}+\gamma_{j}+r_{jh}+c_{jk}+{ø}_{i}+\epsilon_{aikhj}$$

where *y_aikhj_* was the vector of phenotypic value of genotype *a* from population *i* (a column describing which entry is the tested clone or one of two checks (CP00-1101 and CP96-1252) evaluated from the *h^th^* row of the *k^th^* column from the *j^th^* replicate in trial, γ*j* was the effect of *j^th^* complete replicate, *rjh* was the effect of the *h^th^* row and the *j^th^* replicate, *cjk* was the effect of *k^th^* column within the *j^th^* replicate, *ø_i_* is the fixed effect of the *i^th^* population (a column describing which entry is the clone or check, *g_a_* is the random effect term for the *a^th^* genotype with $g_{a}∼N(0,\sigma_{g}^{2})$ and $\sigma_{g}^{2}$ is the variance due to genotype or line effect, and *ϵ_aikhj_* is the residual error with $\epsilon_{aikhj}∼N(0,\;R\sigma_{\epsilon }^{2})$ , **R** is a diagonal matrix accounting for heterogeneous error variance, and $\sigma_{\epsilon }^{2}$ is the population variance of *ϵ_aikh_*. The predict function in ASREML-R 4.0 (Butler et al., 2009) was used to obtain the BLUPs. The variance component estimates from a different model were used to estimate the broad sense heritability (H^2^) following entry mean basis as H^2^ = σ^2^_g_/(σ^2^_g_ + σ^2^_e/R_), where σ^2^_g_ and σ^2^_e_ reflect variance associated with genotype and error, respectively, while R indicates the number of replications. The Pearson correlation coefficients among the five tested RA traits and resulting heat map were conducted in GraphPad Prism 9 software (www.graphpad.com).

### 2.5 Relationship among the clones

An additive relationship matrix was constructed for the 432 clones in the genotypic dataset described above using the AGHmatrix package v2.0.4 in R (Amadeu et al., 2016). Genotypes were assumed to be decaploid (ploidy = 10), but pseudodiploid parameterization was used because the genotypic matrix had only three values (heterozygous, homozygous for reference, and homozygous for alternate). The resulting matrix was centered, and then singular value decomposition was performed. Principal components were extracted and plotted using the plotly graphing package in R (Li and Bilal, 2021).

### 2.6 Genome-wide association study

Multivariate mixed-linear models for the five traits of interest were fit using an Average Information Restricted Maximum Likelihood (REML) algorithm (Gilmour et al., 1995) implemented in ASReml-R v 4.1.0.160 (Butler et al., 2009). The multivariate models took the form:


(5) y=Xb+Zu+e

where ***y*** was a vector of phenotypes from K cropping cycles that is partitioned into ***y’*** = **[*y’*_1_, *y’*_2_, *y’*_3_]** and each vector ***y’_k_*** has length *n_k_* where *n_k_* was the number of individuals phenotyped in cropping cycle *k*; $X={⊕}_{k=1}^{K}\;X_{k}$ , where ***X_k_*** was an incidence vector for each cropping cycle of length *n_k_*; ***b*** *’* = [*b*_1_,*b*_2_,*b*_3_]*’* was a vector of fixed effects for each cropping cycle; $Z={⊕}_{k=1}^{K}\;Z_{k}$ , where ***Z_k_*** was an *n_k_xc* incidence matrix of unique clones *c* in cropping cycle *k.* and ***u’*** = [***u’*_1_, *u’*_2_, *u’*_3_**] was an matrix of random clone effects and each vector ***u’_k_*** has length c; and ***e’*** = [***e’*_1_, *e’*_2_, *e’*_3_**] was a vector of residuals where each vector ***e’_k_*** has length *n_k_*. Clone effects were assumed to follow a multivariate normal distribution with mean zero and an unstructured variance–covariance matrix equal to $U⊗I_{c}$ where *I_c_* was a *C x C* identity matrix, *U* was a K x K matrix in which cropping cycle variances of clone effects $\sigma_{uk}^{2}$ on the diagonal and $\sigma_{u_{kk'}}^{2}$ , the covariance between clone effects in different cropping cycles was on the off-diagonal. Finally, residuals were assumed to be normally distributed with mean zero and correlated among cropping cycled with variance equal to $E⊗I_{k}$ where ***I_k_*** was a *K* x *K* identity matrix and ***E*** was a K x K matrix with residual variance within a cropping cycle $\sigma_{ek}^{2}$ on the diagonal and residual covariance between cropping cycles $\sigma_{e_{kk'}}^{2}$ on the off-diagonal. BLUPs for each clone in each cropping cycle were extracted from the model’s predicted values, and then the RA for each trait was calculated using the equation from Coto Arbelo et al. (2021) as previously described.

GWAS was performed using the R packages GWASpoly v2.10 (Rosyara et al., 2016) for each trait using the RA metric described above using the BLUPs calculated from Equation 5 and the same genomic dataset used throughout the study. A diploid status was used due to insufficient sequence read depth to estimate allele dosage. The polygenic covariance matrix was computed for each chromosome according to the set.K function in GWASpoly. The maximum genotype frequency was set as 0.988 (1-5/N where N = 432). Both additive and dominance models were tested for each trait. The significance thresholds for each model and trait combination were determined for a false discovery rate (FDR) of 0.1 following the method in Storey (2002). Manhattan and qqplots plots were generated using the qqman v0.1.8 package in R. The LD contour plots for 14 most significant associated SNPs (p ≤ 0.0001) was created from genotypic data of the 432 clones of sugarcane population using JMP genomics 10.0 software and physically aligned to the sorghum reference genome.

### 2.7 Identification of candidate genes

Sorghum reference genome was used, since it is the closest diploid organism to sugarcane. Genes physically located nearby the significant associated markers with traits were investigated for their possible annotation and function in the literature. The tag sequences containing SNPs associated with traits of interest were also BLASTed against the sugarcane reference genome “R570” using the genomic resource provided at https://sugarcane-genome.cirad.fr/ and a default e-value of 1e-10.

### 2.8 Genomic selection

For each trait, four different genomic prediction models were evaluated *via* 5-fold cross-validation repeated 25 times. The 5-fold validation was completed by randomly selecting four-fifths of the individuals for the training and the remaining fifth as the validation population. Iteration number was used to set the seed for randomization of genotypes into the test and training sets to ensure the same training set was used to develop all four models. Details of the four models tested, namely, the Ridge regression BLUP [RR-BLUP (Endelman, 2011)], Additive-dominance-epistasis [ADE (Covarrubias-Pazaran, 2016)], Reproducing kernel Hilbert space [RKHS (Gianola and Van Kaam, 2008)), and Bayes A (Perez and De los Campos, 2014), can be found in our previous publication (Islam et al., 2022a). Several evaluation criteria were considered to assess prediction accuracy and model bias: prediction accuracy (Pearson’s correlation coefficient *r* between predicted and observed values), coincidence index (CI) (the proportion of genotypes in the top 20% of observed values that were also in the top 20% of predicted values), and the slope and intercept from a regression of the predicted values on the observed values. For a perfectly unbiased model, the slope and intercept values would be 1 and 0, respectively.

To test the effect of including highly significant GWAS hits as fixed effects in prediction models, the scheme outlined above was repeated for each trait with a slight modification: before fitting each prediction model, a GWAS model testing only additive effects was performed using just the training population from each iteration. GWAS was otherwise performed using the same parameters as described above. The most significant hit was extracted from the GWAS results and the vector corresponding to that SNP from the genotypic marker matrix was included as a fixed effect in the prediction model.

For each trait and model, evaluation metric results for all iterations were summarized in box and whisker plots generated using ggplot v3.3.4.

## 3 Results

### 3.1 Distribution, heritability, and correlation

**Figure 1:**
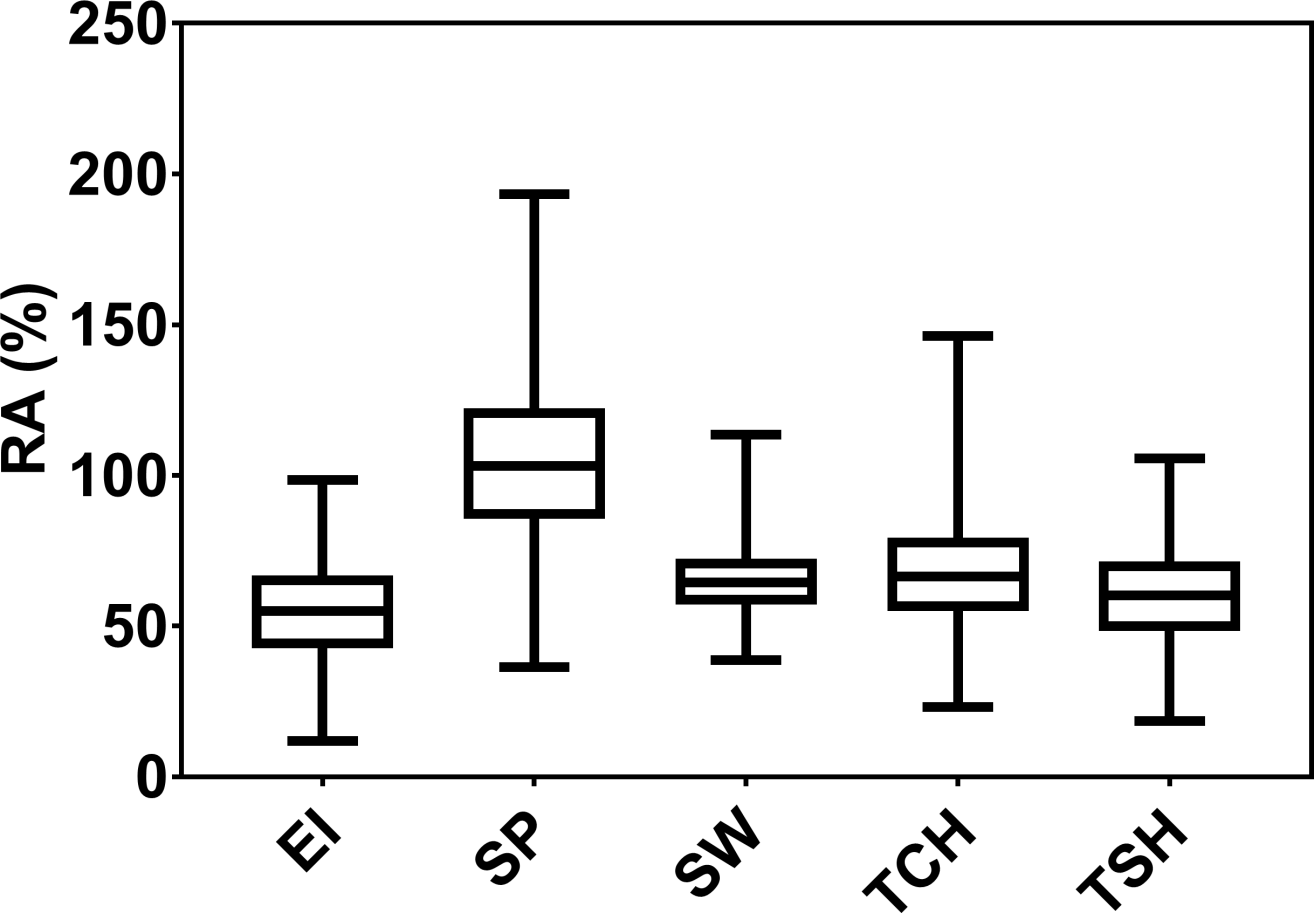
Box plot distribution of the ratooning ability (RA; %) of economic profitability (EI), stalk population (SP), stalk weight (SW), cane yield (TCH), and sucrose yield (TSH).

The RA of sugarcane [RA (%)] for five traits, EI, SP, SW, TCH, and TSH, was estimated. The distribution, mean values, ranges, and variance components of these values are included in **Figure 1** and **Table 1**. The RA for SP appears to be highest over PC performance (average 104.45%, range 23.8%–216.8%), followed by TCH (average 67.5%, range 5.4%–146.3%). Likewise, the genetic variance for SP was highest (477.94) followed by TCH (224.14). The broad-sense heritabilities (H^2^) calculated from variance components were moderate to high (**Table 1**). Once again, the highest heritability for the RA was observed in SP (0.71) followed by TSH (0.66), TCH (0.65), EI (0.62), and SW (0.58).

**Table 1:** The mean, range, variance components, and broad-sense heritability (H^2^) of the ratooning ability (RA; %) of stalk weight (SW), stalk population (SP), cane yield (TCH), sucrose yield (TSH), and economic profitability (EI).

Traits	BLUPs mean	Mean	Max	Min	Genetic variance $\left({\hat{\sigma }}_{g}^{2}\right)$	Residual $\left({\hat{\sigma }}_{e}^{2}\right)$	Heritability (H^2^)
EI	55.48	55.38	99.50	9.83	172.69	210.40	0.62
SP	104.47	104.48	216.84	23.82	477.94	396.11	0.71
SW	65.25	65.13	131.14	28.44	78.04	113.99	0.58
TCH	67.67	67.49	146.32	15.38	224.14	242.30	0.65
TSH	60.67	60.47	105.64	14.90	168.40	176.49	0.66

A mixed linear model estimated the best linear unbiased prediction (BLUP), variance components, and heritability (entry mean basis).

**Figure 2:**
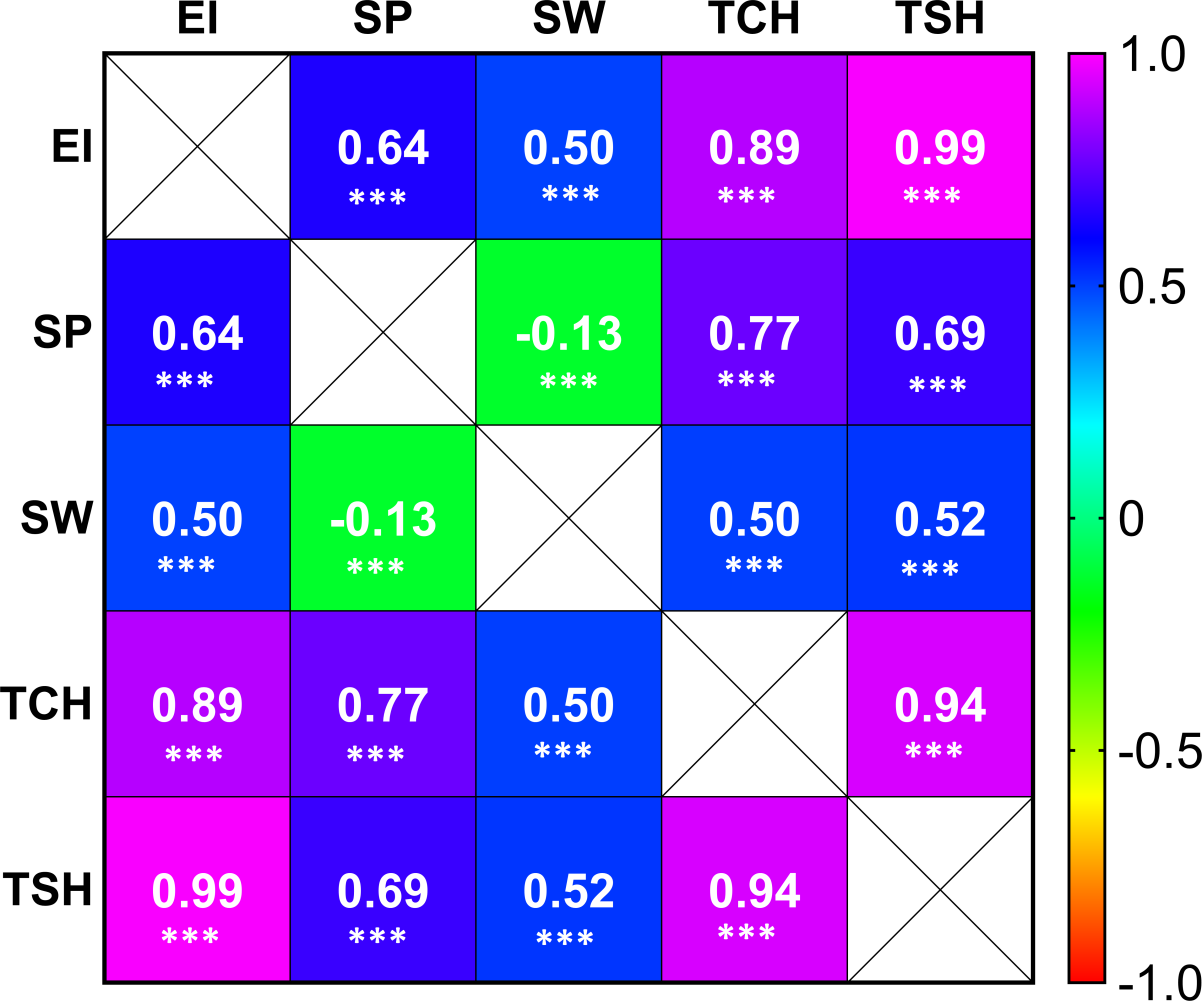
Correlation coefficient heat map among the tested traits [ratooning ability (RA; %) of stalk weight (SW), stalk population (SP), cane yield (TCH), sucrose yield (TSH), and economic profitability (EI)]. The three asterisks (***) denote the correlation coefficient (R) significant at p ≤ 0.001.

Highly significant correlations were observed among the five tested RA traits in sugarcane (**Figure 2**). As expected, the highest positive correlation coefficient (0.99) was observed between EI and TSH, while the lowest negative correlation coefficient (-0.13) was associated between SP and SW. The RA of SP has a higher correlation coefficient value than that of SW with the other three tested traits (TCH, TSH, and EI).

### 3.2 Population structure

**Figure 3:**
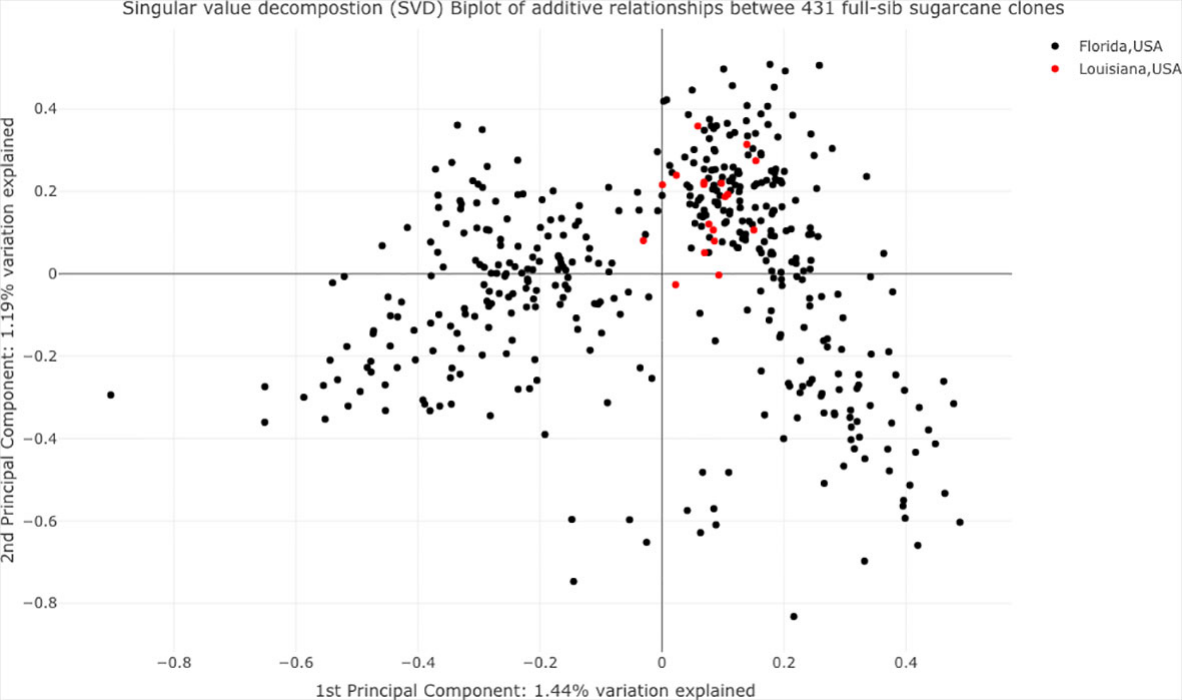
Principle component analysis of 432 tested sugarcane clones. The black and red dots represent clones belonging to the sugarcane breeding programs at Canal Point, Florida, and Houma, Louisiana, USA, respectively.

A principal component biplot was created using the SNP data to examine the population structure among the tested clones (**Figure 3**). Based on the pseudo-diploidized SNP data, we found little evidence for population structure among the tested clones; the first two principal components explained 1.44% and 1.19% of the total variation, respectively. The tested clones did separate into two groups based on first principal components. One group fell in both the third and fourth quadrants and the majority of the tested CP clones and almost all of the Louisiana clones were placed in this cluster.

### 3.3 Marker–trait association

**Figure 4:**
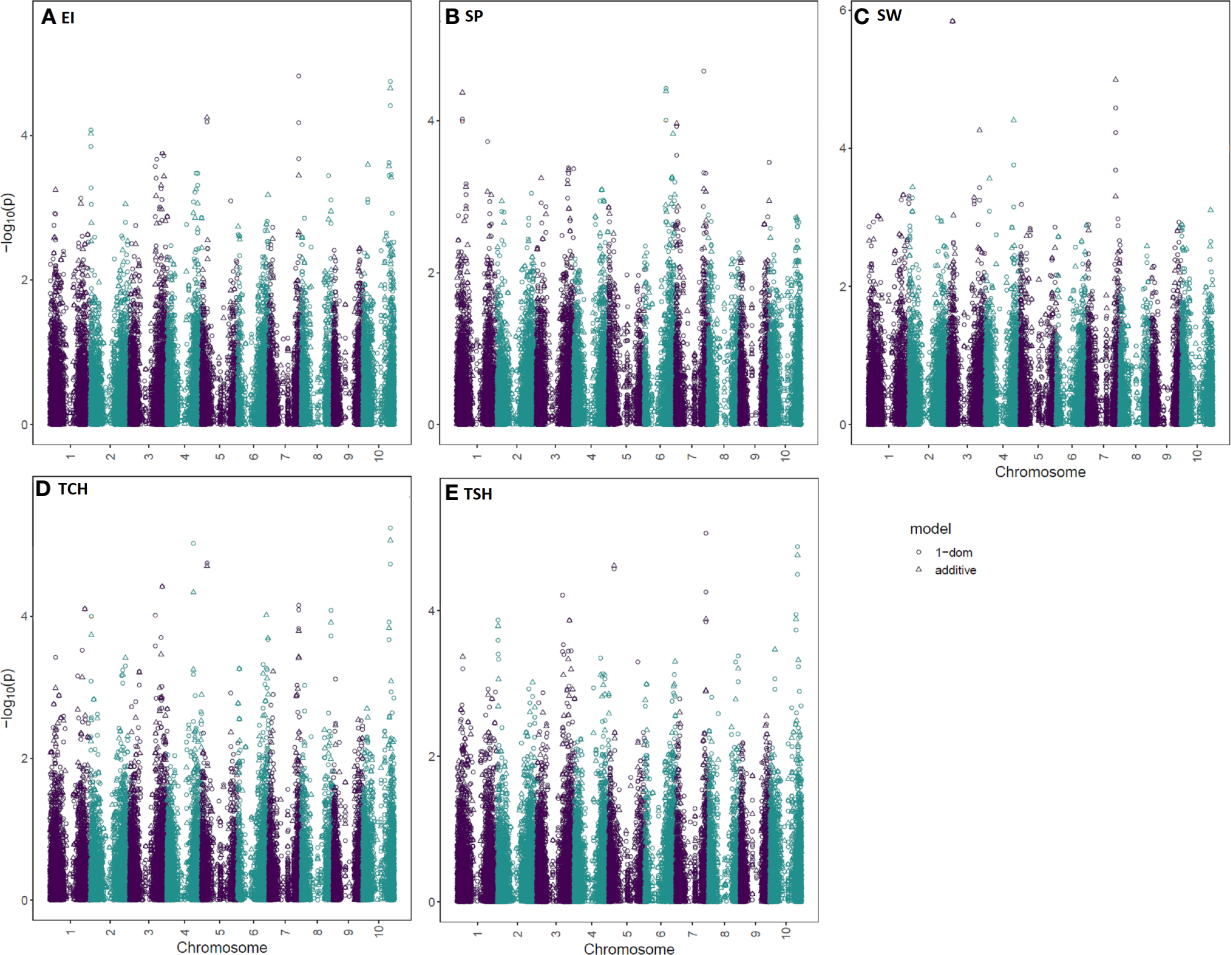
Manhattan plots for the sugarcane ratooning ability (RA; %) of economic profitability (EI), stalk population (SP), stalk weight (SW), cane yield (TCH), and sucrose yield (TSH) using two models (additive and 1-dominant). The negative log_10_ converted p*-*values were plotted against the marker positions on the 10 chromosomes of the sorghum reference genome.

The RA of the five traits (EI, SP, SW, TCH, and TSH) were subjected to GWAS analysis and results of marker–trait associations were included in **Figure 4**, **Table 2**, and **Supplementary Table S2**. Three GWAS models (additive, 1-dominant reference, and 1-dominant alternative) were explored, and FDR-adjusted p-values were determined for each SNP. Adjusted p-values greater than 0.001 are reported in **Supplementary Table S2** with an indication of whether they are statistically significant according to a genome-wide FDR of 0.1.

**Table 2:** Summary of marker–trait associations and quantitative trait loci (QTLs) detected at different threshold levels of p-values for the ratooning ability of five traits in sugarcane.

Trait^a^	Marker–trait association				QTL^b^			
	p ≤ 0.001	p ≤ 0.0001	Total	# Chr.	p ≤ 0.001	p* *≤ 0.0001	Total	# Chr.
EI	17	5	22	8	3	0	3	3
SP	15	3	18	5	2	0	2	2
SW	14	4	18	6	1	0	1	1
TCH	24	11	35	9	5	1	5	5
TSH	17	5	22	9	2	0	2	2
Total	87^c^	28^c^	115^c^	9^c^	13	1	13	5^c^

^a^ Traits are economic index (EI), stalk population (SP), stalk weight (SW), cane yield (TCH), and sugar yield (TSH).

^b^ Marker loci mapped within 5-Mb intervals in the same chromosome were considered as a putative QTL.

^c^ Some marker loci were associated with more than one trait.

Since many observed p-values deviated from the uniform distribution of expected and observed probability of obtaining an association with the respective traits, the quantile-quantile (Q-Q) plots demonstrated a potential for false-positive associations. Results revealed that most of the observed p-values follow a uniform distribution, but the few in linkage disequilibrium (LD) with a causal polymorphism have significant p-values in the tail (**Supplementary Figure S1**). A total of 14 significant marker–trait associations were detected for the RA of the five tested traits (**Table 2**). Of the 14 associations, two, four, five, and three were linked with traits EI, SW, TCH, and TSH, respectively. No significant marker–trait association was found for SP. There were eight nonredundant SNPs associated with all tested traits. One SNP (S10_53811870), located on sorghum chromosome 10, was commonly associated with three traits, EI, TCH, and TSH. Two SNPs located on sorghum chromosomes 5 and 7 (S05_10359269 and S07_59440142) were significantly associated with two traits: TCH, TSH and EI, TSH, respectively. One SNP, S03_8476715, located on sorghum chromosome 3, was significantly associated with SW for all three models tested.

A single putative QTL was considered when multiple loci associated with a trait were within a 5-Mb interval. The QTL nomenclature was according to McCouch et al. (1997). A total of six QTLs associated with the RA of the four tested traits (EI, SW, TCH, and TSH) were identified (**Table 2**; **Supplementary Table S3**). The highest number (three) of QTL was associated with the RA of TCH and located on three different chromosomes. The extremely significant (p value ≤ 0.000001) loci (*qRATCH-cS10*) associated with the RA of TCH comprises three SNPs (*S10_51068605, S10_53811870*, and *S10_55391413*) covering 4.3 Mb on chromosome 10. The SNP (*S10_53811870*) with the strongest association with the RA of TCH (p value = 8.55E-6) is located at position 53,811,870 bp on chromosome 10.

### 3.4 Candidate genes

**Figure 5:**
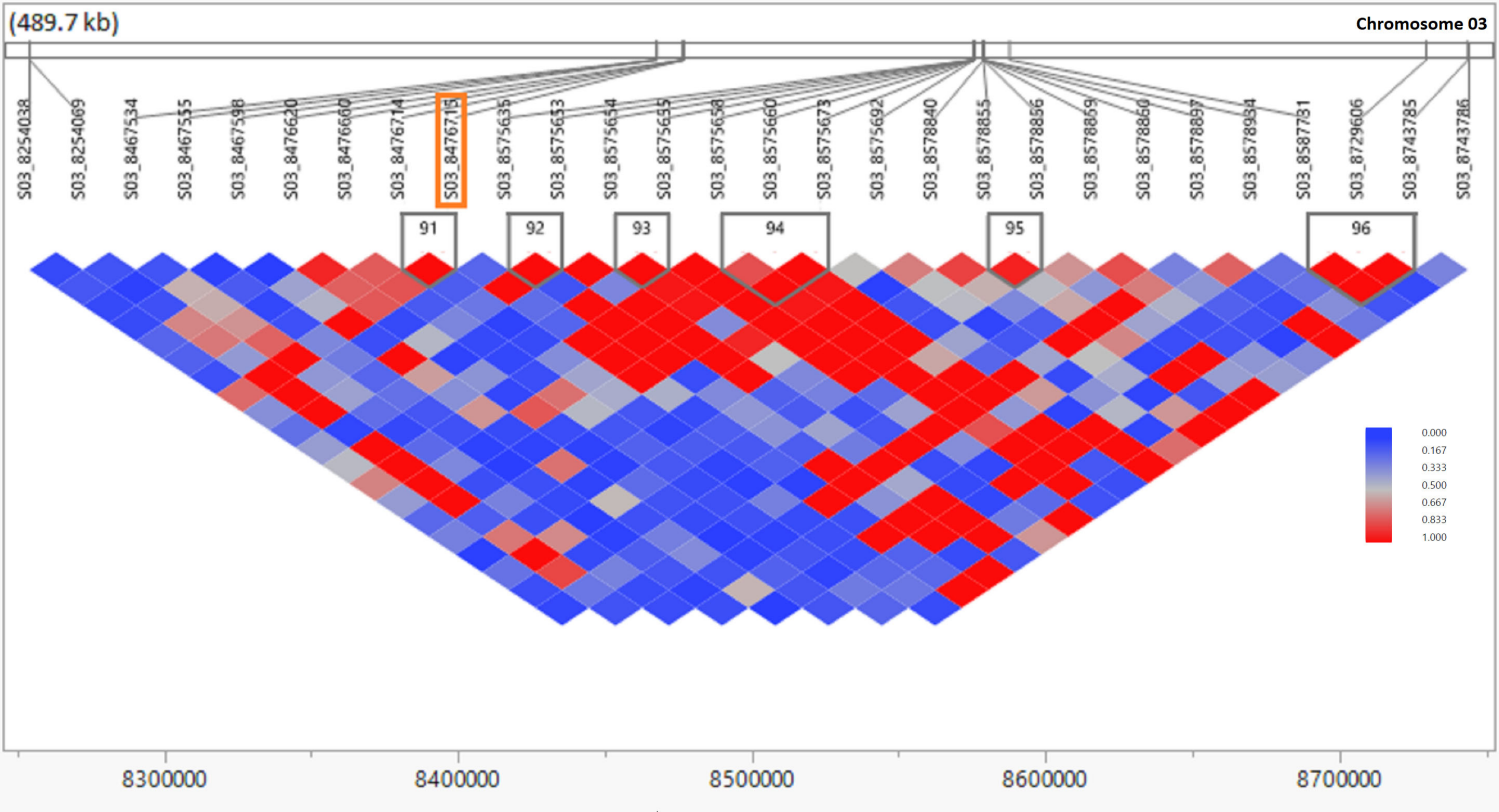
Linkage disequilibrium (LD) observed in the genomic region of the most significant SNP (p ≤ 0.00001) associated with ratooning ability of stalk weight (SW) in sugarcane. The LD contour plot is physically located in the sorghum genomic region of 8.2–8.8 Mb on chromosome 03. The LD contour was created from genotypic data of the 432 clones of the sugarcane population using JMP genomics 10.0 software. The X axis is physical distance in Mb and r^2^ (CorrCoeff) between marker pairs is shown in different color blocks as per legend. The most significant SNP (S03_8476715) is indicated inside the orange box.

SNPs significantly associated with the RA of sugarcane were aligned with the sorghum genome, and those SNPs (7) with closely located genes (<20 kb from the SNP) were included in **Table 3** and **Supplementary Table S4**. We also BLASTed the tag sequences of those seven SNPs against the monoploid sugarcane “R570” genome (Garsmeur et al., 2018) using an e-value cutoff of 1e-10, and associated homologous genes were listed in **Supplementary Table S4**. Gene annotation of the sorghum genome suggested that seven putative genes were colocated with those significant SNPs associated with the five RA traits in sugarcane. The high proportion of genes located 0.0 kb from significant SNPs indicates that many SNPs occurred within the coding sequence of genes. Out of seven genes, the annotation of five genes were found to have homologs among *Arabidopsis*, rice, and “R570” (sugarcane monoploid genome) gene families and function. The LD contour plot near the most significant SNP (*S03_8476715*) associated with the RA of SW showed that there are several small LD blocks within a large one, indicated by red color in the plot and most of the SNPs have linked each other as indicated by the strong LD (**Figure 5**). This SNP is colocated with gene Sobic003G096000 in the sorghum reference genome, which is functionally annotated as aldolase superfamily protein. The LD contour plots for the other 14 significant SNPs also exhibited similar results (**Supplementary Figure S2**).

**Table 3:** Candidate genes associated with significant single-nucleotide polymorphisms (SNPs; p ≤ 0.0001) linked with the ratooning ability (RA; %) of economic index (EI), stalk population (SP), stalk weight (SW), cane yield (TCH), and sugar yield (TSH) along with their annotation from the sorghum reference genome.

SNP	Chr.	Candidate gene (Sobic. #)	Distance from SNP (kb)	Gene annotation	Trait
S03_64270241	3	003G314700	0.0	Vps51/Vps67 family (components of vesicular transport) protein	EI, TCH, TSH
S03_8476715	3	003G096000	0.0	Aldolase superfamily protein	SW
S04_51880407	4	004G168200	0.0	heat shock protein 101	TCH, TSH
S05_10359269	5	005G079201	4.8	F-box/RNI-like superfamily protein	EI, TCH, TSH
S07_56501497	7	007G137300	3.7	Homeodomain-like superfamily protein	SW
S07_59440142	7	007G160050	0.0	dicarboxylate carrier 2	EI, TCH, TSH
S10_53811870	10	010G195000	0.0	SEC7-like guanine nucleotide exchange family protein	EI, TCH, TSH

### 3.5 Genomic selection prediction

**Figure 6:**
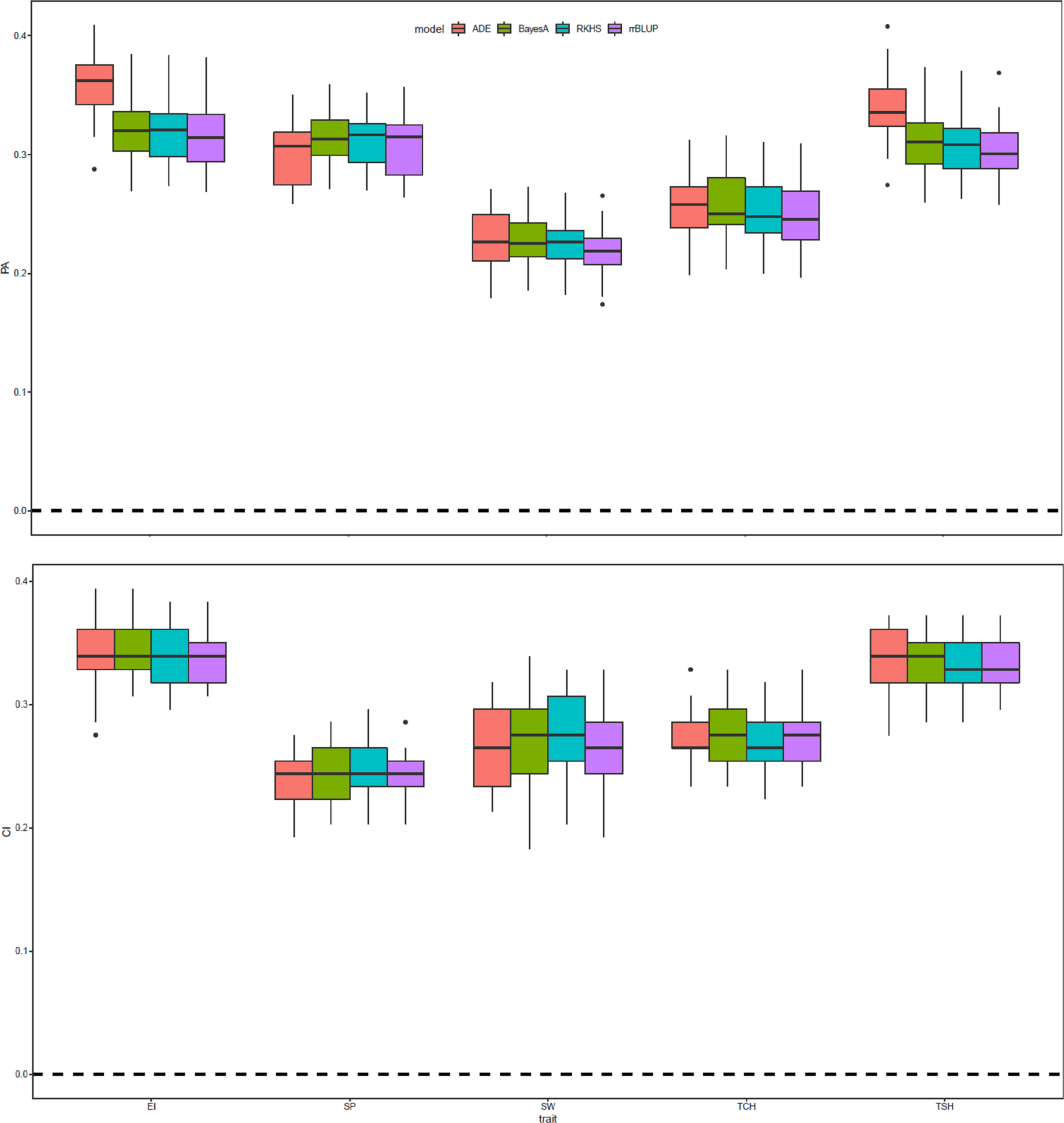
Prediction accuracy (PA) and coincidence index (CI) of the genomic estimated breeding value (GEBV) of sugarcane ratooning ability (%) of economic profitability (EI), stalk population (SP), stalk weight (SW), cane yield (TCH), and sucrose yield (TSH) for 5-fold cross-validation (5-fold CV) of four genomic selection (GS) methods with and without most significant peak marker from genome-wide association analysis as a fixed covariate for the respective traits. The 5-fold CV was completed by randomly selecting four-fifths of the individuals for the training and the remaining fifth as the validation population.

The prediction accuracy for the five RA traits was evaluated using four GS methods with and without highly significant SNPs as fixed effects (total eight models), and four metrics were used to evaluate the GS models (prediction accuracy, CI, slope, and intercept). Results of genomic prediction and CI for the RA of EI, SP, SW, TCH, and TSH are presented in **Figure 6**. The overall prediction accuracies attained for EI were greater than that of the other tested traits for the RA. The highest and lowest accuracies (0.36 and 0.21) were observed for EI with the ADE model without fixed effects and SW with RR-BLUP model with fixed effect, respectively. In general, the standard genomic prediction models that did not include significant markers as fixed-effect covariates yielded the highest prediction accuracies; however, the differences in prediction accuracies between models were slight and not statistically significant in any case. Slope and intercept of the regression lines were used to measure the prediction bias of the models. By comparing between the slope and intercept distribution for standard models and models, the results showed that models with fixed effects were severely deviated from the expected values of 1 and 0, respectively (**Supplementary Figure S3**).

**Figure 7:**
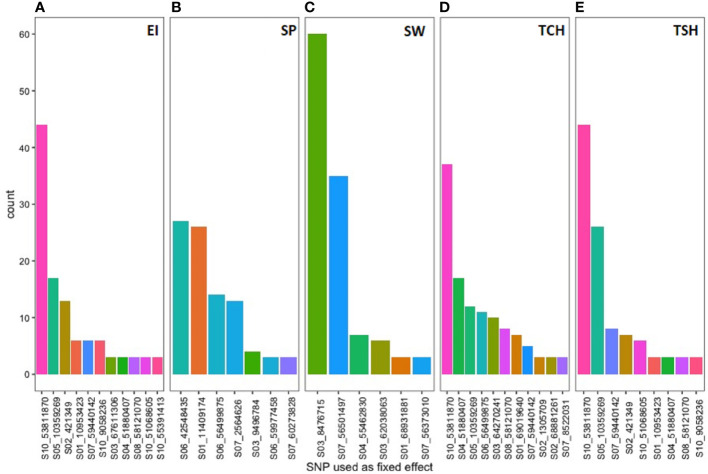
The single-nucleotide polymorphism (SNP) markers associated with the most significant QTL linked to tested ratooning ability traits [(**A**) EI, **(B)** SP, **(C)** SW, **(D)** TCH, and **(E)** TSH] and their frequency were used as a fixed effect in the prediction models.

The name and frequency of SNPs utilized as fixed effect in the four GS models for the five RA traits were included in **Figure 7**. The SNP *S03_8476715* located on sorghum chromosome 3 was used most frequent 60 times as fixed effect during predicting the RA of SW followed by *S10_53811870* SNP located on chromosome 10 was used as fixed effect for predicting the GEBV of three (EI, TCH, and TSH) RA traits. Both the SNPs showed significant association during the GWAS analysis using GWASpoly in this study.

## 4 Discussion

The profitability of sugarcane production is highly dependent on the RA of sugarcane cultivation. A genomic analysis of the sugarcane RA for the five traits was conducted through a combined GWAS and GS approach. GWAS results discovered a total of 14 significant marker–trait associations. A total of seven putative candidate genes associated with the RA in sugarcane were identified. Thus, the findings of the genomic analysis conducted in this study provide new information and a potential avenue for improving the RA traits in sugarcane through molecular breeding.

Worldwide, the sugarcane industry is facing increased planting costs and shortages of laborers. Thus, improvement of the RA is one of the most important target traits in breeding because replanting costs can be reduced by planting varieties that have a high RA, thereby increasing profitability. There are three main methods that have been reported to calculate the RA in sugarcane (Dunckelman, 1982; Milligan et al., 1996; Zhou and Shoko, 2012). Although those methods have some differences and performed differently, we measured the RA following the methods described by Milligan et al. (1996) and Coto Arbelo et al. (2021), since it is easiest to calculate. The box plot distribution of phenotypic data of all five RA traits was continuous (**Figure 1**) and normal, signifying that several QTLs likely control the inheritance of those complex traits. Although the traits that contribute to the RA are very complex, broad-sense heritability estimates were moderate to high for each trait studied, which is congruent with previous studies (Todd et al., 2017; Abu-Ellail et al., 2019). As expected, the RA for SW was negatively correlated with the SP. This is because clones with heavier stalks tend to produce fewer tillers in the ratoon crop. The correlation coefficients SP with TCH, TSH, and EI were higher than that of SW. This implies that SP is the greatest contributor to the RA of sugar and yield traits in sugarcane. These results are consistent with few previous studies (Zhou and Shoko, 2012; Coto Arbelo et al., 2021).

The molecular and genetic basis of the RA variation has received little attention in scientific literature. Thus, it has been unknown what molecular mechanism accounts for sugarcane’s ability to ratoon. To break through this impasse, we have conducted GWAS analysis for the five RA traits, detected putative candidate genes, and explored GS for the first time. In GWAS, mutations and potential functional markers statistically associated with complex quantitative traits are identified as underlying causal genes, mutations, and genetic variants (Islam et al., 2016). However, our efforts were not easy and further complicated for several reasons. At first, estimating the RA is complicated by difficulty in accounting for genotype by year interactions. Using multivariate mixed modeling in conjunction with the methods described by Milligan et al. (1996) and including data from two ratoon cycles, we estimated the RA of five traits while accounting for covariance between ratoon crops. Like all other genomic studies in sugarcane, our study faced numerous challenges such as a large genome size (10 Gb), complex genome architecture, types and level of ploidy, method of propagation, highly heterozygous genetic makeup, and nonadditive genetic variation. When we began this study, a sugarcane genome reference was not openly available. Therefore, we aligned our sequence data to the sorghum genome, likely missing genomic regions unique to the sugarcane genome. Furthermore, while software packages such as GWASpoly are designed to model the effect of allelic dosage in polyploids, doing so requires sufficient sequence read depth to estimate ploidy. Given the read depth of our sequence data, we modeled sugarcane as a pseudo-diploid. We used GWASpoly, an R package, to quantify three different types of polyploid gene action, including additive, simplex dominant, and duplex dominant (Rosyara et al., 2016; Yang et al., 2019b). By using three models (additive, 1-dom-alternate, and 1-dom-reference), eight nonredundant SNPs were found significantly associated with four RA traits (EI, SW, TCH, and ISH) (**Figure 4**, **Table 2**; **Supplementary Table S2**). Out of six putative unique QTLs associated with the RA of the five tested traits, one QTL located on 10 was associated with three traits (EI, TCH, and TSH). This is not surprising, since EI and TSH were estimated from TCH data as well as highly correlated with each other (**Figure 2**). Thus, these three traits are interdependent and closely related genetically, suggesting that the improvement of one trait will improve the others, since they share common QTLs. The outcome of these results could not be compared, since no other study has been reported. Thus, all of the marker–trait associations and QTLs related to the RA identified in this study are believed to be novel.

The probe sequences of the significant seven associated markers with the RA of the five traits were utilized to pinpoint the putative candidate genes possibly regulating the RA in sugarcane. Sugarcane stubble morphology studies have found that deep (long) roots, a large number of buds, a large number of live buds, and a large number of permanent roots (Chumphu et al., 2019), along with a reasonable leaf size and a reasonable tillering ability (Gomathi et al., 2013), are associated with a strong RA. Photosynthetic parameters such as chlorophyll fluorescence and stomatal conductance associated with ratoon sugarcane yield are significantly correlated with these variables (Chumphu et al., 2019). A C4 plant, such as sugarcane, has superior photosynthesis over a C3 plant because it concentrates CO_2_ around Rubisco and uses the NADP malic enzyme to increase the sugar yield (Faralli and Lawson, 2020). The most significant associated SNP (*S03_8476715*) with the RA of SW is physically colocated with the gene Sobic.003G096000 in the sorghum reference genome, which was annotated as an aldolase superfamily protein (**Table 3**; **Supplementary Table S4**). Surprisingly, this SNP was also most frequently picked up as a fixed-effect covariate during genomic prediction analysis. When the probe sequence of this SNP was compared with the sugarcane monoploid genome “R570” using BLAST, it was aligned with a similar homologous gene (**Supplementary Table S4**). This nuclear encoded chloroplast gene regulates several pathways that produce energy through photosynthesis, glycolysis, gluconeogenesis, and the Calvin cycle in the plant (Lu et al., 2012; Mininno et al., 2012; Carrera et al., 2021). It also reported that this gene governs root growth through helping the transportation of metabolites into the root (Mininno et al., 2012). An additional critical SNP associated with several target traits (*S07_59440142*) with the RA of EI, TCA, and TSH was linked with gene *Sobic.007G160050* and *Sh07_t012030* in the sorghum and sugarcane monoploid “R570” reference genome, respectively. These genes are functionally annotated as dicarboxylate carrier 2 and regulate the transportation of metabolites such as malic acid and abscisic acid (ABA) in the cell for controlling several energy-related pathways glycolysis, gluconeogenesis, and the TCA cycle in the plant (Narsai et al., 2011; Shingaki-Wells et al., 2014; Barreto et al., 2022). It has been found that ABA regulates the withering of ineffective tillers through molecular signals in the form of hormonal interactions in sugarcane (Qiu et al., 2018). Another significant SNP (*S10_53811870*) associated with three RA traits (EI, TCH, and TSH) and picked up most frequently as a fixed-effect covariate during genomic prediction was linked with sorghum gene *Sobic.010G195000*. This gene is actively participating in plant development and growth. It has been shown that a double mutant of this type of gene reduced plant growth specially root length (Suo et al., 2021). It may be possible to gain greater insights into the mechanisms that underlie genetic control of the RA in sugarcane by further investigating these genomic locations and genes.

GS was experimentally evaluated in this study using four different GS methods in conjunction with the GWAS results incorporated as fixed effects to determine the prospect for future sugarcane hybrid breeding. The RA of five yield and sugar traits (EI, SP, SW, TCH, and TSH) were evaluated, and 5-fold cross-validated prediction accuracy differed by traits and models. The overall prediction accuracies were moderate (0.21–0.36). We are unable to compare our results, since there are no GS reports published on the RA traits. However, prediction accuracies on other yield, sugar, and disease traits in sugarcane were found to be low to moderate in several previous reports (Gouy et al., 2013; Hayes et al., 2021; Islam et al., 2021; Yadav et al., 2021; Islam et al., 2022a). The nonparametric model ADE performed slightly better than other models for some traits. Studies have found that nonparametric models performed better in the presence of dominant and epistatic effects, reducing prediction error and increasing prediction accuracy while detecting SNP–SNP and SNP–covariate interactions (Lubke et al., 2013; Waldmann, 2016). Hence, our results suggest that the genetic makeup of the complex RA traits in sugarcane contains nonadditive effects in this testing population (Howard et al., 2014; Li et al., 2018). Another study in sugarcane also advised that additive and nonadditive gene effects play a big role during predicting the GEBV in the GS study (Hayes et al., 2021). Overall, the models fitting fixed effects for the most significant associated markers for each respective trait performed poorer than the standard models without fixed effect, although this difference was not statistically significant. The genetic architecture of the tested traits has a greater effect on the performance of a fixed-effect model during genomic prediction (Bernardo, 2014; Rice and Lipka, 2019). Models that include QTL with large effects, such as those for disease resistance, tend to improve the prediction accuracy (Poland and Rutkoski, 2016) while including fixed marker effects for complex traits controlled by many small effect QTL reduces the performance of genomic prediction and increases model bias. A fixed-effect covariate included in the validation set may offer little or no advantage over a standard model in terms of prediction accuracy, as it may have a substantially weaker association in the validation set (Rice and Lipka, 2019).

This is the first study of its kind for the RA, and while prediction accuracies are low to moderate, these are likely good enough to begin testing GS practically for the RA. There is promise on the ability to increase prediction accuracies by addressing some of the known issues in marker development and genome complexities as mentioned above, as the heritability of the five RA traits is quite high. The findings of this study lay the groundwork for opening a new avenue for improving RA of sugar- and yield-related traits in sugarcane through breeding.

## Data availability statement

The genotype data presented in the study are deposited in the Ag Data Commons repository : https://doi.org/10.15482/USDA.ADC/1528986.

## Author contributions

MI contributed to the conceptualization, data curation, formal analysis, funding acquisition, investigation, methodology, project administration, resources, supervision, visualization, writing—original draft, and writing—review and editing. KC contributed to the formal analysis, software, visualization, and writing—review and editing. PM contributed to the conceptualization, resources, and writing—review and editing. AH-K contributed to the writing—review and editing and resources. AL contributed to the writing—review and editing. All authors contributed to the article and approved the submitted version.
