# Supplementary material for: A first look at the ability to use genomic prediction for improving the ratooning ability of sugarcane
Source: Front Plant Sci. 2023 Aug 2;14:1205999. doi: 10.3389/fpls.2023.1205999 (PMC10433174; doi:10.3389/fpls.2023.1205999)

Figure S1. QQ-plot of sugarcane ratooning ability of ratooning ability (%) of stalk weight (SW), stalk population (SP), cane yield (TCH), sucrose yield (TSH), and economic profitability (EI) using different models (additive, 1- dominant alternate, and 1- dominant reference). Chromosomes (1-10) are based on the *Sorghum bicolor* reference genome.
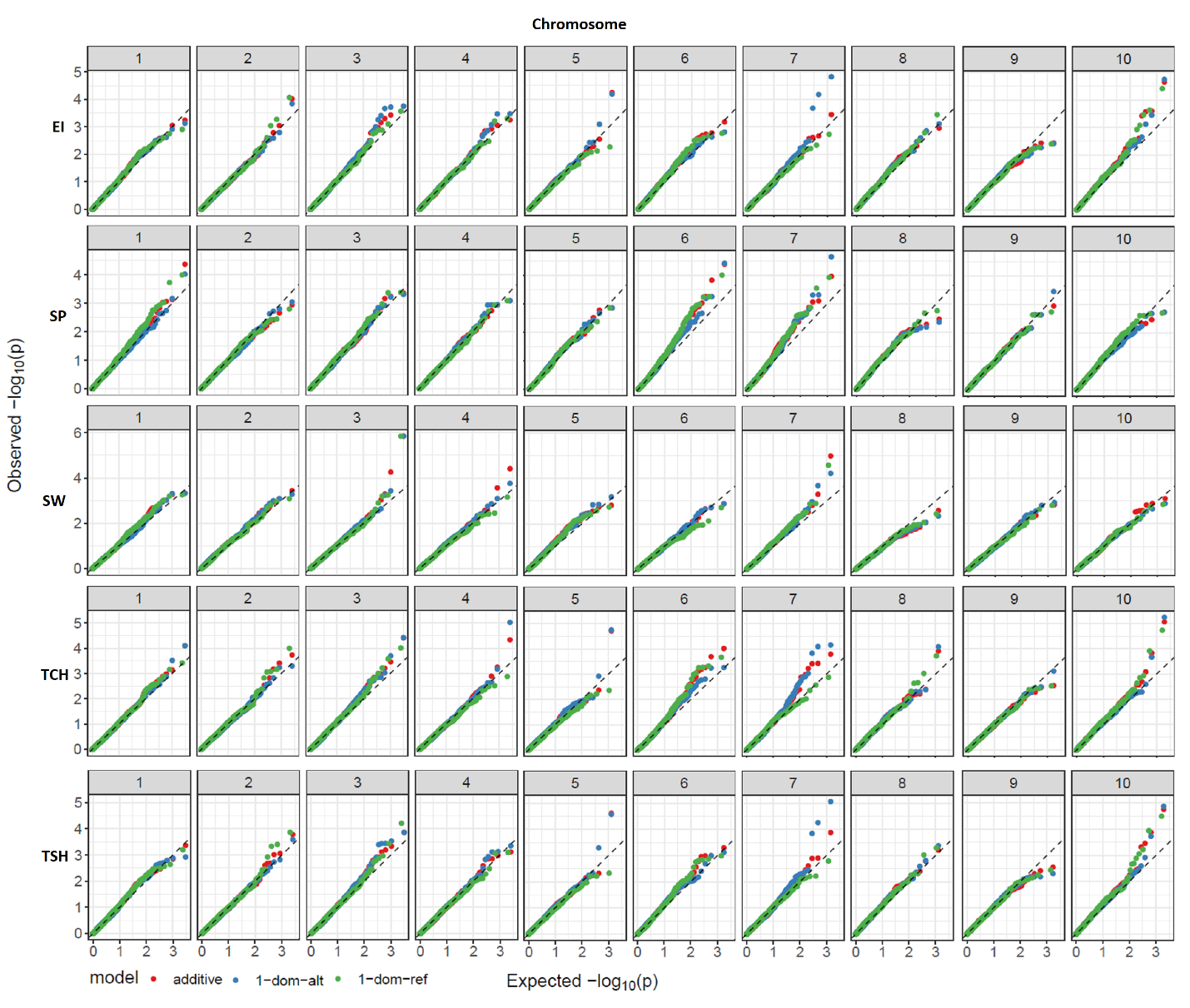


Figure S2. Linkage disequilibrium (LD) observed in genomic regions of significant SNPs associated with ratooning ability of five traits in sugarcane. The LD contour plot is physically located in the sorghum genomic region of nine different chromosomes. LD contour plots were created from genotypic data of the 432 clones of sugarcane population using JMP genomics 10.0 software. X axis is the physical distance in Mb and r^2^ (CorrCoeff) between marker pairs shown in different color blocks as per legend. The most significant SNPs are indicated inside the orange boxes.


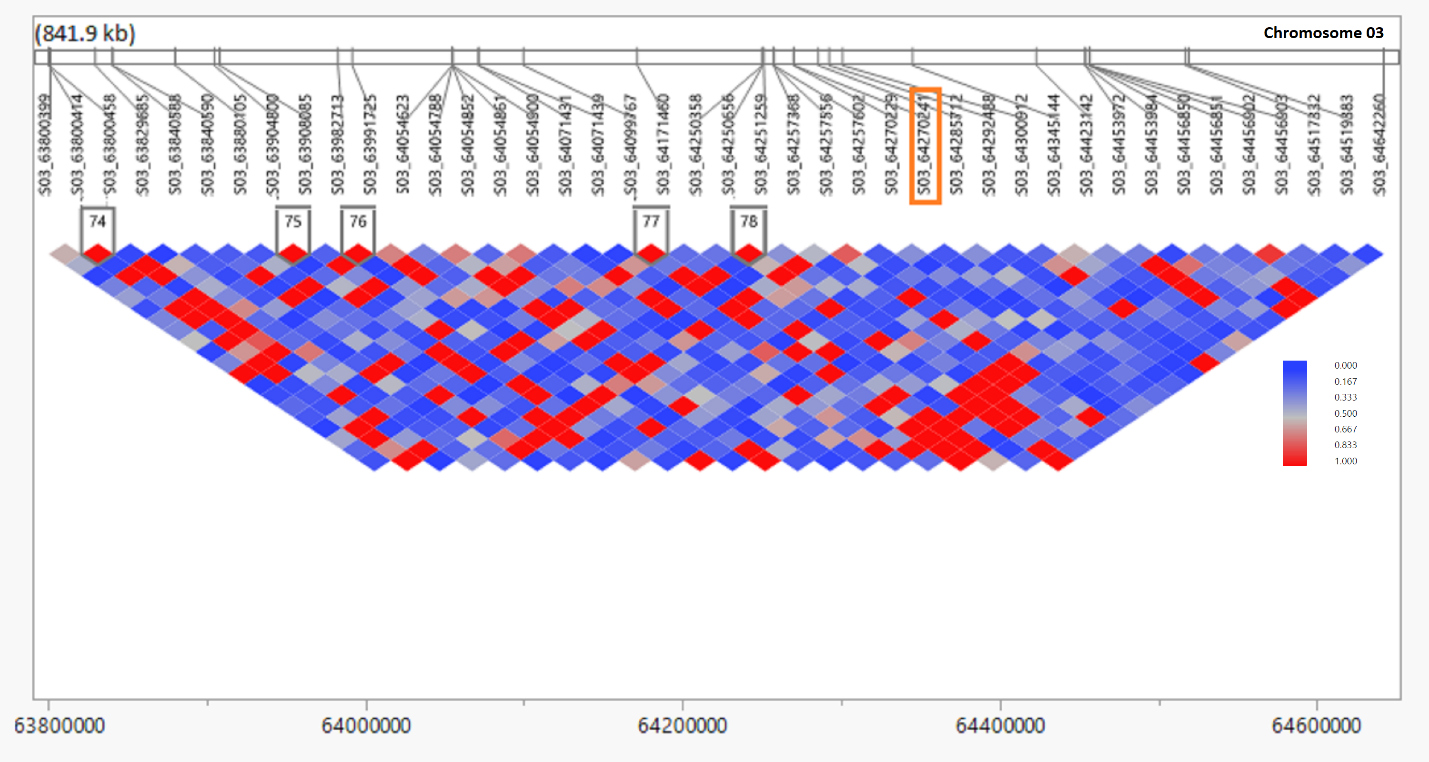

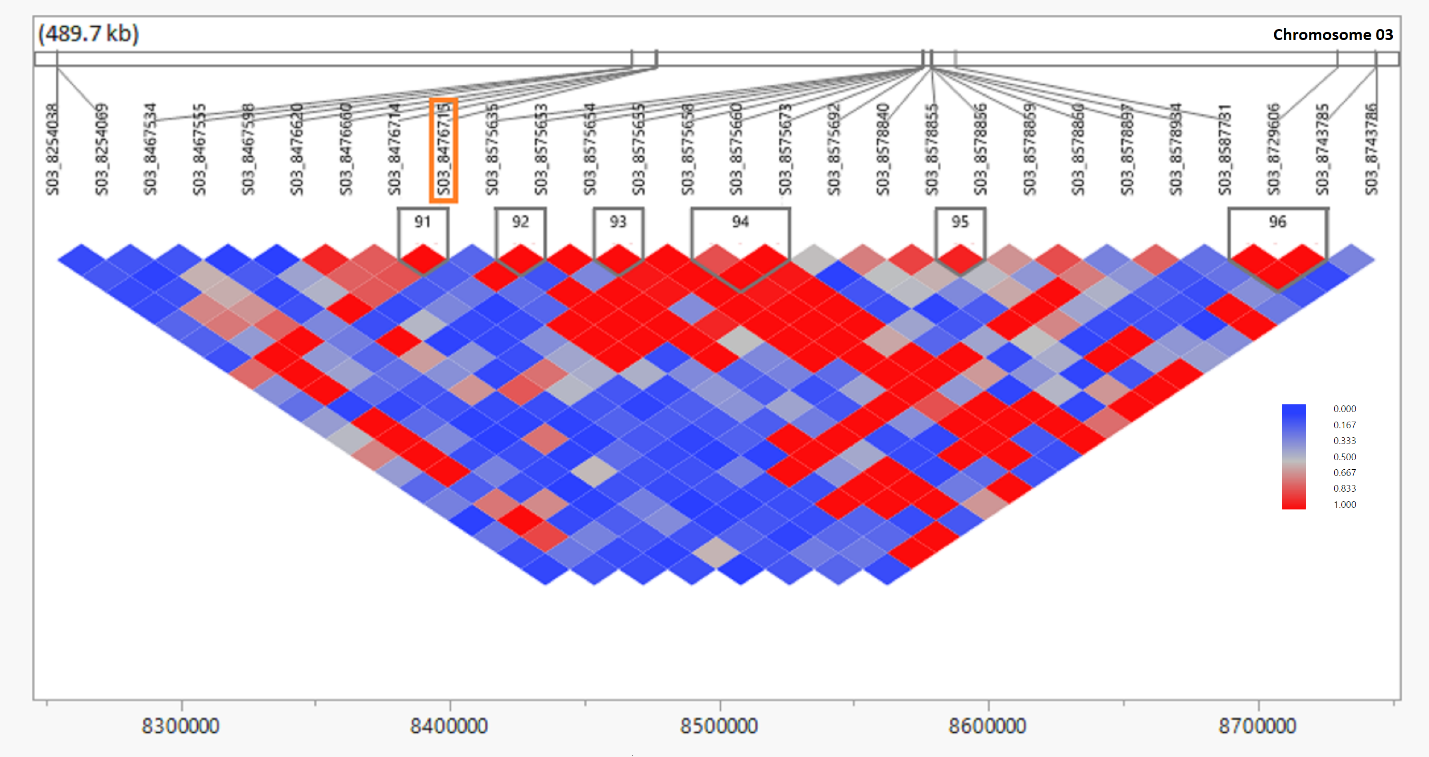

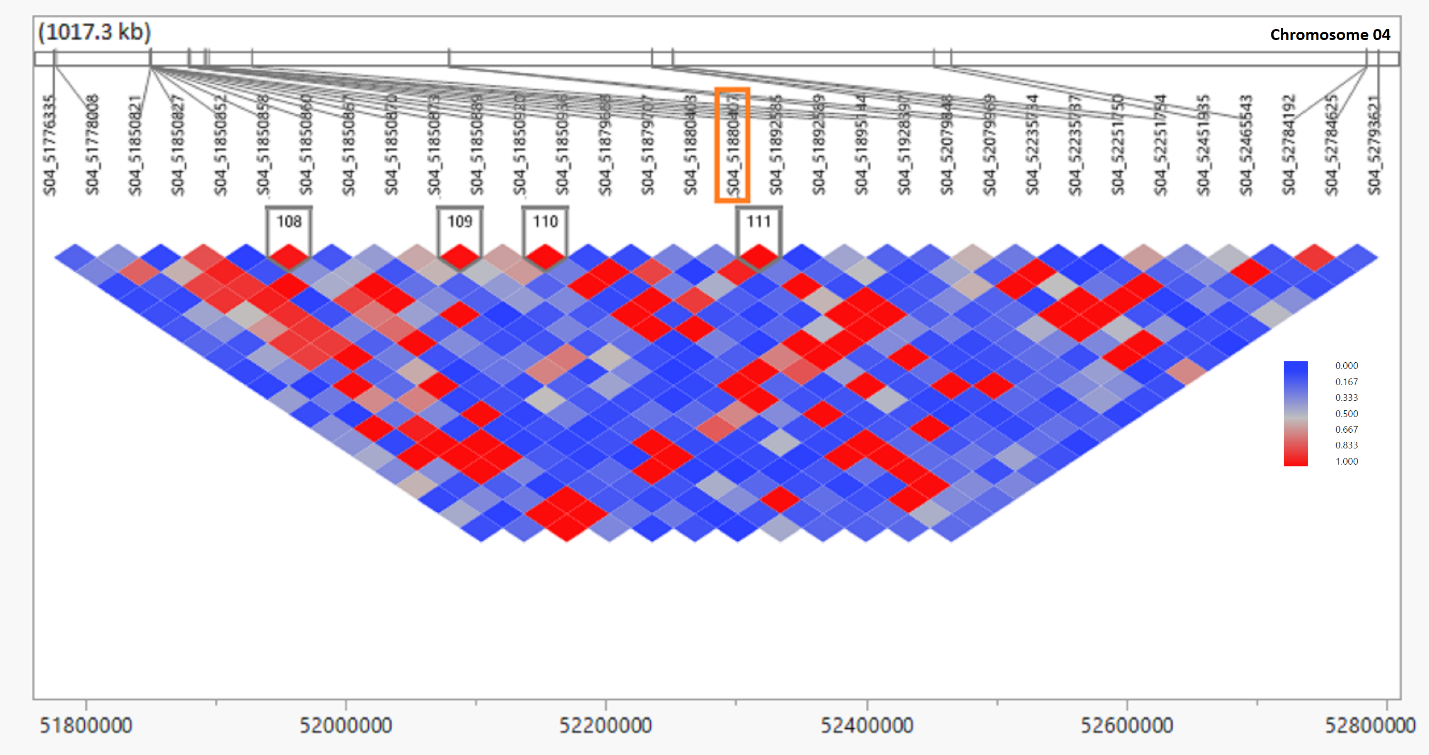


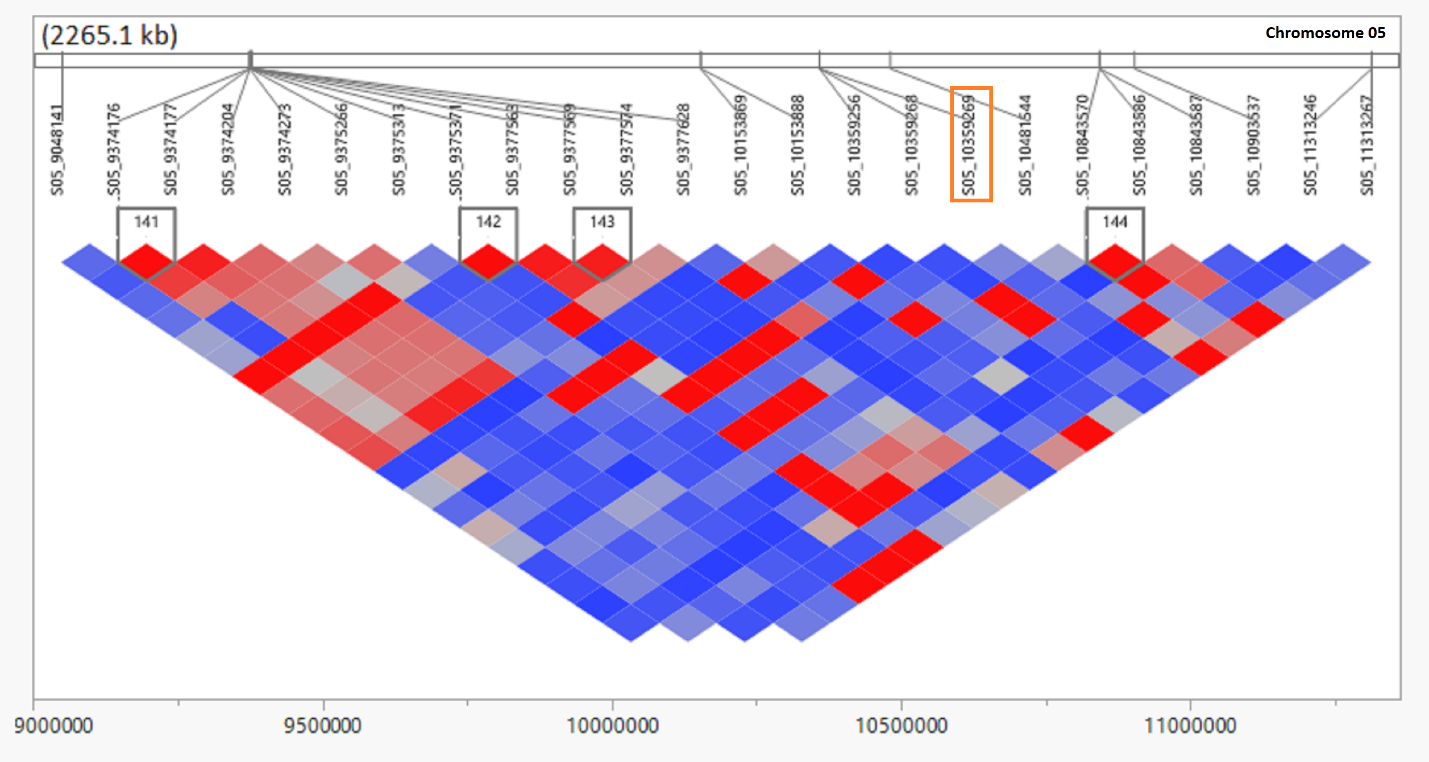

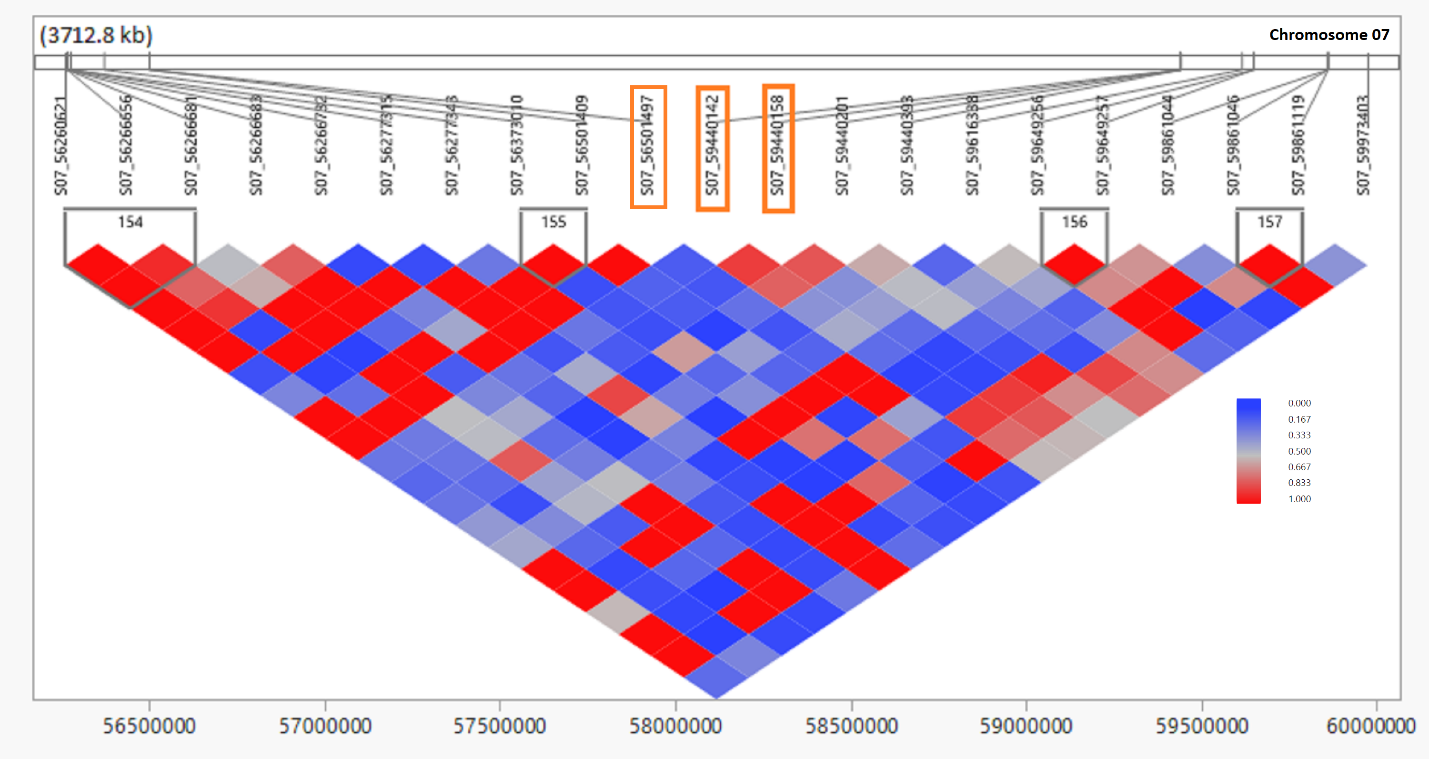

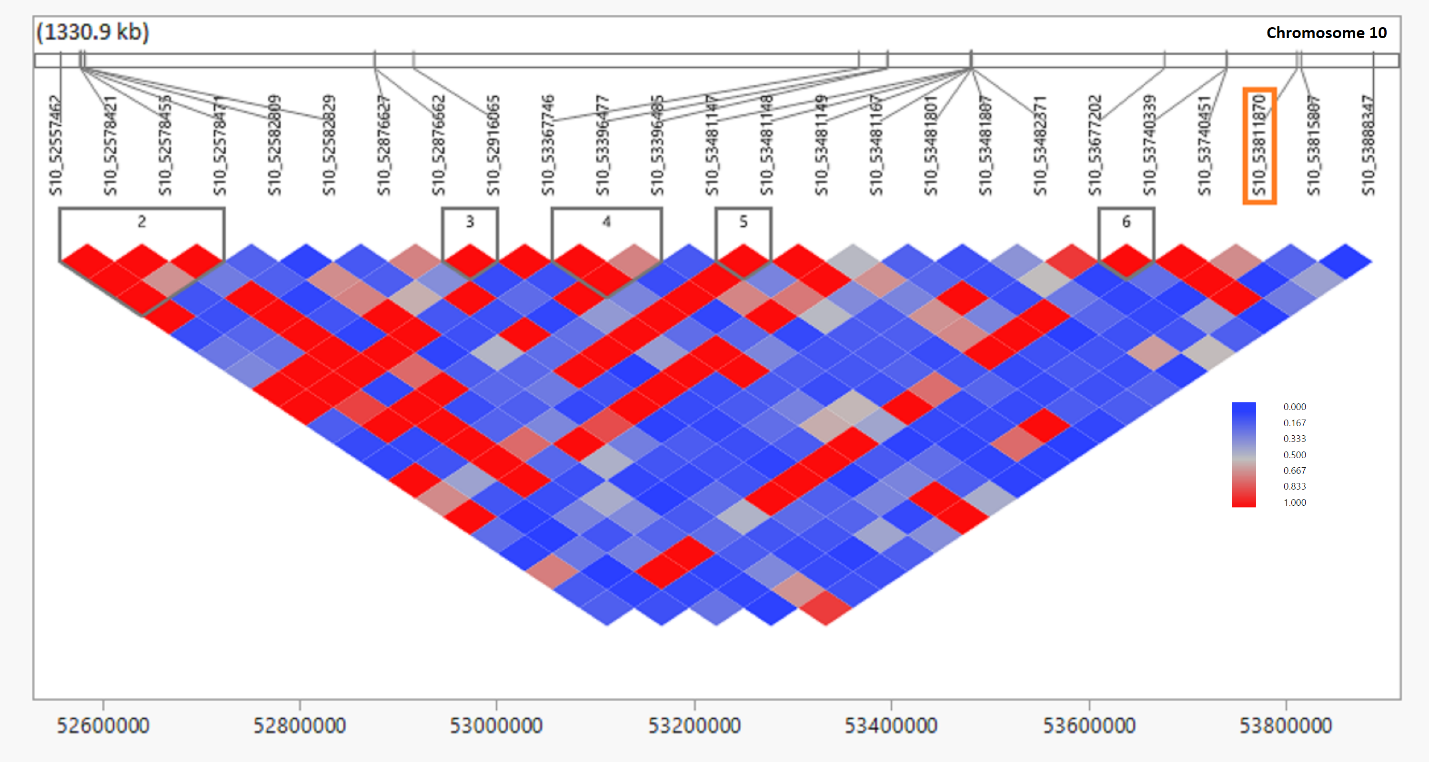


Figure S3. Intercept and slope estimated through regression analysis between the genomic estimated breeding estimated value (GEBV) and phenotype value (BLUP) of sugarcane ratooning ability (%) of economic profitability (EI), stalk population (SP), stalk weight (SW), cane yield (TCH), and sucrose yield (TSH).


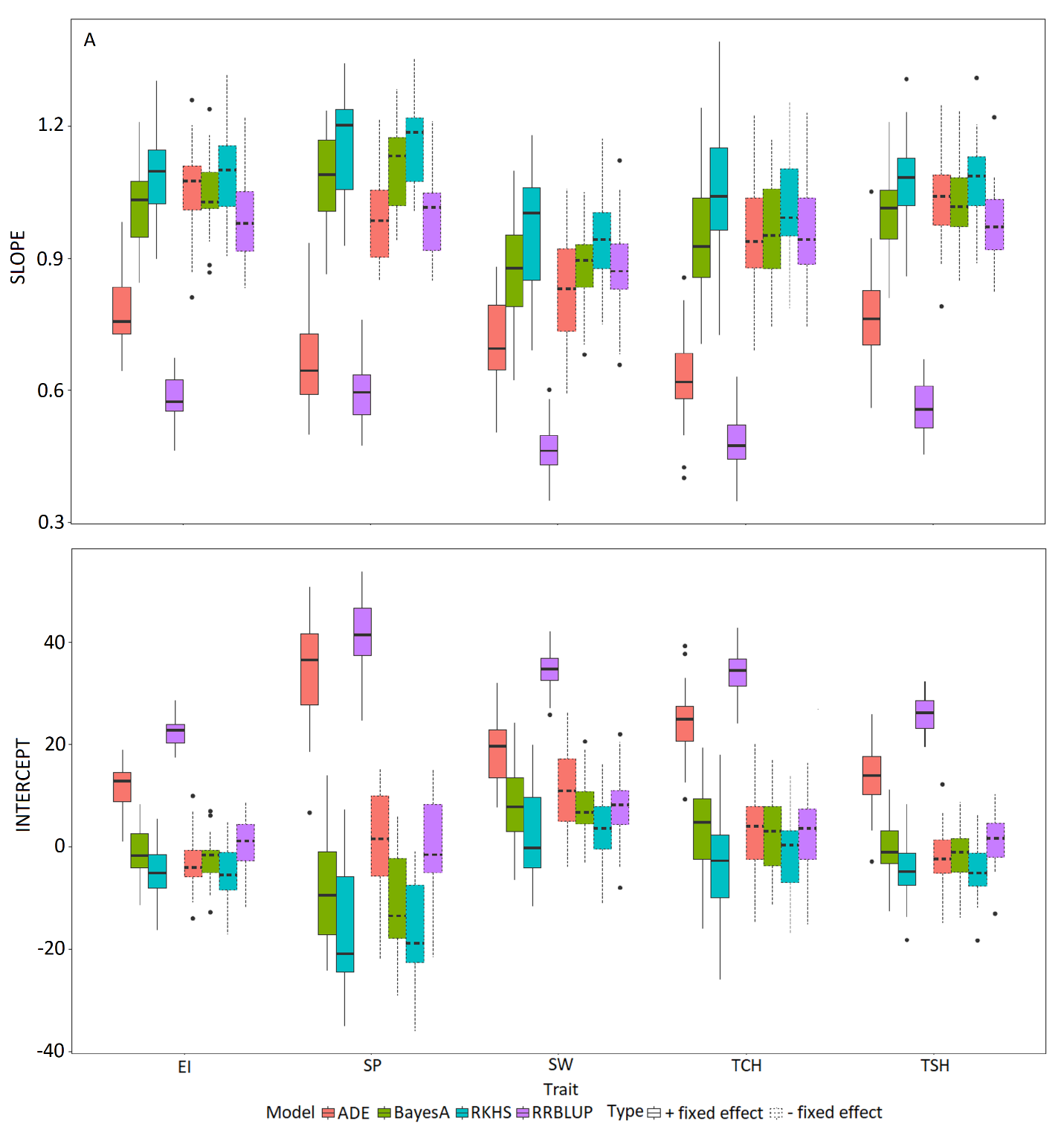

Supplement: Supplementary file 5 [file DataSheet_1.docx]
